# Healing architecture and Snoezelen in delivery room design: a qualitative study of women’s birth experiences and patient-centeredness of care

**DOI:** 10.1186/s12884-020-02983-z

**Published:** 2020-05-11

**Authors:** Jane Hyldgaard Nielsen, Charlotte Overgaard

**Affiliations:** 1 Department of Midwifery, University College of Northern Denmark, Selma Lagerløfs Vej 2, 9220 Aalborg Øst, Denmark; 2 Public Health and Epidemiology Group, Department of Health Science and Technology, Aalborg University, Niels Jernes Vej, 14, 9220 Aalborg Øst, Denmark

**Keywords:** Hospital design, Birth environment, Birth experience, Patient-centered care, Qualitative methods, Semi-structured interviews

## Abstract

**Background:**

The physical place and environment has a profound influence on experiences, health and wellbeing of birthing women. An alternatively designed delivery room, inspired by the principles of healing architecture and Snoezelen, was established in a Danish regional hospital. These principles provided knowledge of how building and interior design affects the senses, including users’ pain experience and stress levels. The aim of the study was to explore women’s experience of the environment and its ability to support the concept of patient-centeredness in the care of birthing women.

**Methods:**

Applying a hermeneutical-phenomenological methodology, fourteen semi-structured interviews with low-risk women giving birth in an alternative delivery room at an obstetric unit in Denmark were undertaken 3–7 weeks after birth.

**Results:**

Overall, women’s experiences of given birth in the alternative delivery room were positive. Our analysis suggests that the environment was well adapted to the women’s needs, as it offered a stress- and anxiety-reducing transition to the hospital setting, at the same time as it helped them obtain physical comfort. The environment also signaled respect for the family’s needs as it supported physical and emotional interaction between the woman and her partner and helped relieve her concern for the partner’s well-being. The psychosocial support provided by the midwives appeared inseparable from the alternative delivery room, as both affected, amplified, and occasionally restricted the women’s experience of the physical environment.

**Conclusion:**

Our findings support the use of principles of healing architecture and Snoezelen in birth environments and add to the evidence on how the physical design of hospital environments influence on both social and physical aspects of the well-being of patients. The environment appeared to encompass several dimensions of the concept of patient-centered care.

## Background

Childbirth is an important life experience for women involving a number of interrelated psychological and physiological processes, which are influenced by the social, organizational and environmental context [1]. The woman’s overall birth experience is an important outcome of labor [2, 3]; the quality of this experience thus affects the future well-being and health of the woman and the baby, and the relationship with her partner [2, 4]. A positive birth experience is associated with long-lasting benefits for the woman, including improved self-esteem and empowerment important to her role as a new mother [1, 4, 5]. A negative birth experience, on the other hand, may have a lifelong psychological impact in the form of post-partum depression [6], PTSD symptoms [7], increased fear of childbirth [8] and possible impact on breastfeeding [9] and mother and child relationship [10]. The overall experience of childbirth is influenced by the course of labor, complications, pain experiences, support, sense of control, as well as the woman’s birth expectations [2, 4]. However, as argued by e.g. Fahy and Parratt [11], the physical birthplace and the delivery room play an important role in the woman’s birth experience.

The shift of place of birth from home to hospital in most high- and middle-income countries means that the design of delivery rooms is generally dominated by a medical paradigm [12]. Such an environment for birth has been associated with both disempowerment and a lack of accommodation to women’s psychological needs [13]. Hospital design improvements have been shown to affect neurological and physical responses and to have the potential to alter patients’ state of mind [14]. The physical birth environment is known to affect the production and release of neurochemicals, such as oxytocin – a key mediator of social and emotional behavior [15], as well as the physiology of normal birth [13]. Women’s sense of safety and satisfaction with the birth experience may therefore be significantly influenced [16].

The world over, a variety of alternative maternity care settings have been designed to support normal labor and birth, as well as to stimulate more positive birth experiences. Organization models as well as care and staffing models vary greatly, but their differences notwithstanding, such alternative birth environments all aim to decrease birthing mothers’ anxiety and promote mobility and personal control [12]. The alternative birth environments are therefore guided by a common philosophy that delivery rooms need not look like conventional hospital rooms [12]. Establishing a physical environment to support the women and their families’ activities could help them toward a caring, effective, and safe birth experience [12, 14], an attribute of more patient-centered healthcare practices [17].

In line with general recommendations [17, 18] patient-centered care is a marker of quality in maternity care and health service delivery in general, as this is documented to increase patient satisfaction. An alternative birth environment may minimize negative healthcare impacts and have a positive effect on women’s perception of patient-centered care and likewise on the ability to meet future generations’ needs for health and social care.

An alternative delivery room inspired by the principles of healing architecture and Snoezelen was established in a Danish hospital, drawing on knowledge of how building and interior design affects the senses, including users’ pain experience and stress levels [12, 14, 19, 20]. The effect of the alternative delivery room on the maternal and perinatal birth outcomes was evaluated in a randomized, controlled trial (RCT) (To be published).

This qualitative study is a separate but adjunct study to the RCT, and the objective was to gain deeper understandings of women’s experiences of the alternative birth environment and its ability to support the concept of patient-centeredness in the care of birthing women. The obtained knowledge may help health care managers, professionals, organizations, and management strengthen the quality of maternity healthcare.

## Methods

### Design

Identifying the experiences of giving birth at the alternative delivery room from womens’ perspectives required a qualitative approach focusing on the lived experiences of women. The Hermeneutic phenomenological research method underpinned this study, as it attempts to unveil the world as experienced by the subject through their life world stories [21, 22]. The philosophical and methodical approach is refined by great scholars like Martin Heidegger and Hans George Gadamar [21, 22]. Research reality is perceived as individual constructions in response to different situations and the epistemology is grounded on the belief that it is possible to gain knowledge through subjective experience and insights [21]. Hermeneutic phenomenological research relies on reflection which is often mentioned as the researcher use of empathy or relevant prior experience as an aid to data analysis and/or interpretation of meanings [22].

This study was based on individual, semi-structured interviews with women who had given birth at the alternative delivery room. This design is often used when evaluation and deeper understanding of specific interventions in health care service are needed as it enables researchers to explore the experiences, intentions, and actions of the individual, as well as the impact of the experience [23].

#### Presuppositions and preunderstandings of researchers

It is important for the researcher to be reflective about the ways in which their questions, methods and subject position might impact on data [22]. In this study, the researchers presuppositions and understandings of the phenomenon were colored by a shared background in the midwifery profession and based upon a fundamental belief that birth experiences can permeate women’s’ lives and that positivity in relation to labor and birth should be supported. This allowed the researchers to be open to the women’s experiences, and in interpretation of data, to pursue the methodological principles of the hermeneutic movement; awareness of one’s own understanding, putting one’s understanding into play and put themselves in someone else’s place [23].

#### The concept of patient-centered care

Patient-centered care is seen as key to achieving quality in health care [14, 17, 18]. Drawing on Gerteis, Edgman-Levitan, and Daley [24], the data collection, analysis, and interpretation of data focused on the concept of patient-centered care, allowing for a comprehensive understanding of the women’s experiences to unfold. Reflection to which extent the data were supported or differed from the concept of Patient-centered care will be included in the discussion. The concept of Patient-centered care is widely used in different health care setting, and Gerteis, Edgman-Levitan, and Daley’s definition is visualized in Table 1 below.

**Table 1:** Seven dimensions of patient-centered care, inspired by Gerteis, Edgman-Levitan, and Daley [24]

***Respecting patients’ values, preferences, and needs*** Subjective quality of life, needs, autonomy, and sense of well-being	***Physical comfort*** Pain management, activities, and hospital environment
***Coordination and integration of clinical care*** Clear delegation of responsibility, effective communication between health team members, and administrative and organizational support	***Information, communication, and education*** Giving women knowledge about and clear understanding of status, progress, prognosis, processes of care, and self-care options
***Transition and continuity*** Understanding of planning of care, supporting resources, and relevant information at discharge	***Emotional support and alleviation of fear and anxiety*** Relief of anxiety regarding care and treatment and impact on self and family
***Involvement of family and friends*** Support, accommodation, encouragement, and recognition of role and needs of family	

### Setting

The study setting was an obstetric unit at a regional hospital in Denmark caring for approximately 2500 births yearly. The alternative delivery room was opened in January 2015 [25]. As seen in Table 2, it differed from traditional birth environments on several parameters.

**Table 2:** Key characteristics of alternative and standard delivery rooms

	Alternative delivery room	Danish standard delivery room
**Guiding focus of the physical birth environment**	Promoting feelings of well-being, freedom, and control. A safe and normal birth with minimal intervention	Medical safety
**Visual and auditory stimuli**	Snoezelen-inspired audial and visual scenery on three walls providing positive distractions	The woman may bring own sound device
**Interior, furniture, and equipment**	Nordic contemporary style furniture resembling private home environment	Traditional hospital furniture and equipment, including lounge chair
	Traditional hospital labor bed and necessary equipment covered or placed less visibly	Traditional hospital labor bed as central feature of room
	Bathtub	Bathtub may be available
	Relaxation area with sofa bed, chairs, and coffee table	
**Privacy**	Single occupancy, private bathroom	Single occupancy, often private bathroom
**Light**	Overhead light off unless needed for assessment purposes	Overhead light controlled by staff, usually on unless the woman is sleeping
	Dimly lit scenario projections controlled by woman and her partner	
	Dimmed light in relaxation area	

Further information on the background of the alternative delivery room and the RCT can be found in the Study protocol for the RCT. Visualization of both the alternative delivery room and a standard delivery room in Denmark are also to be found here [25].

### Participants and recruitment

The women included in the RCT received both oral and written information to prepare them for the possibility that they might be approached for participation in interviews. The inclusion criteria of low risk women in the RCT, also applied to this adjunct study: Danish speaking, age above 18 years, nulliparous with a spontaneous onset of labor and a baby in a head-down position, and gestational age 37–42 + 0.

We adapted a purposeful sampling strategy to identify and select information-rich cases and individuals especially knowledgeable about the phenomenon of interest [26]. In attempts to achieve a diverse sample which could allow us to identify important patterns across variations [26], we also looked at factors likely to affect women’s experience of labor and birth, e.g., age, marital status, educational background, and complications developed during labor and birth [27].

On this basis, 17 of the women included in the RCT who had given birth vaginally in the alternative delivery room were contacted by phone, and again informed about the study purpose, thus ensuring that participation in interviews was voluntary and based on fully informed consent. Fourteen women accepted the invitation (Table 3), all of them were Caucasian of origin and had their partners present during labor and birth. As the alternative delivery room is a part of the obstetric care unit, all women in this qualitative study gave birth in the alternative delivery room despite that some of the women experienced complications e.g. prolonged labor, post-partum bleeding, influenced heartrate of child and perineal injuries during birth.

**Table 3:** Key characteristics of participants

Woman	Age	Highest degree or educational level achieved	Marital status	Birth complications
**1**	27	Bachelor	Married	
**2**	24	Bachelor	Cohabiting	Yes
**3**	25	Bachelor	Cohabiting	
**4**	26	Bachelor	Cohabiting	
**5**	31	Master	Cohabiting	Yes
**6**	29	Upper secondary	Cohabiting	Yes
**7**	29	Master	Cohabiting	Yes
**8**	26	Bachelor	Married	Yes
**9**	25	Bachelor	Cohabiting	
**10**	28	Bachelor	Married	
**11**	25	Master	Cohabiting	
**12**	28	Upper secondary	Cohabiting	Yes
**13**	29	Bachelor	Married	Yes
**14**	28	Bachelor	Cohabiting	Yes

To allow time for physical and mental recuperation, the interviews were undertaken 3–7 weeks after birth. Offered the choice of interview setting, the women all opted for their own home.

### Data collection

All 14 interviews were conducted by the first author between March and July 2016. An interview guide with open-ended questions aiming to explore the participants’ experience of giving birth in the alternative birth environment was developed on the basis of the literature and field observations as recommended [28].

Although the first interview was intended as a test of the interview guide, its data were included in the study material as the positive dynamics and the interviewee’s spontaneous descriptions of further perspectives and experiences gave no reason for changing the interview guide or further test interviews. Interviews were conducted until the point of saturation, which entails that interviews yield little new knowledge [28]. In general, the interviews allowed the interviewer to gain comprehensive insight into the women’s experiences. All interviews were audio recorded.

### Data analyses

The interviews were transcribed verbatim, as it enables a consistent presentation of the participant experiences [28]. The transcripts were imported into NVivo qualitative data analysis software (QSR International Pty Ltd. Version 11, 2010) for further analysis.

For a deeper understanding of the empirical phenomena, we applied the integrated hermeneutical analytical strategy described by Dahlager and Fredslund, which included a four-step process, permitting the researcher’s preunderstandings to interact with the research process without compromising the data [23]. Even though hermeneutical analysis do not wish or consider it possible for the researcher to be free of own preunderstanding in interpretation of empirical data, the first steps of this analytical strategy was phenomenologically inspired. This allowed the researcher to work with empirically sensitivity and focus on the appearance of the phenomena while gaining an overall first impression of the women’s experience in the reading of the transcribed interviews and while coding meaningful units [23]. After revision, these units were categorized by a single headline describing its essence and then organized thematically. A comprehensive and recontextualized understanding of the women’s experiences was then achieved through interpretation and reflection on the themes. This was done while taking our own preunderstandings, the actual context, and the theoretical contributions into consideration [23].

## Results

### Analysis

The central meaning of the women’s experiences was analyzed and formulated into sub-themes and assembled into three main themes: Emotional support, Involvement of family and Physical comfort (Table 4). The concept of Patient-centered care was used to inform our analysis and the construction of the main themes. The three main themes and subthemes are shown below.

**Table 4:** Main themes, sub-themes

Themes	Sub-themes
Emotional support	Feeling welcome
	Midwife and room inseparability
Involvement of partner	Feeling equal
	A space for the partner
Physical comfort	Positive distractions
	Capturing the room

### Themes

#### Emotional support

##### Feeling welcome

On the whole, women reported an immediate feeling of being welcome on first entering the delivery room. Typical of their experiences, a woman described her sense of emotional support:

… I kind of felt ... love behind it. Yes, I thought, okay ... do they really put so much effort in for us coming here? It gave me a great feeling of being embraced … (Informant 10).

When describing such feelings, the women made associations to a recognizable home-like environment. “Feeling welcome” appeared to be closely associated with the women’s experience of emotional support, comfort and the reduction of the stress and uncertainty experienced in connection with admission for birth. None of the women in our study expressed experiences of stressful elements or limitations induced by spoken and unspoken rules governing the room. The physical environment seemed to support the women’s experience of being a vital part of the organization they now entered, which is especially important if a patient-centered approach is to be supported [24]. Moreover, it seemed to make the women adapt easily to the room and to the transition from home to hospital.

##### Midwife and room inseparability

As in all low-risk births in Denmark, the care was led by a certified midwife, who attended to both medical and psychosocial care needs. All the participants received one-on-one care. The relationship with the midwife, and her ability to provide emotional support, appeared highly significant in all the women’s birth experiences. In most cases, a positive relationship was established between the midwife and the woman, and the experience of the environment appeared to depend on both this relationship and the physical features. The data show that the women’s experiences of the physical environment was affected, amplified, and sometimes restricted by the quality of the relationship with the midwife. As the alternative birthing environment also seemed to influence the midwives’ practices and engagement with the couple, the effect of the midwife and the room appeared inseparable.

Many of the women in our study emphasized the midwife’s ability to small talk in a way that involved their own life perspective and experience. This seemed to strengthen the relationship between the couple and the midwife, as it was interpreted as a sign of equality. For instance, one woman said:

…Well, she wasn’t just sitting there writing, or only present when it was to do with the birth ... she could also talk about other things – that was super nice. Because it also makes her a little more human. It was simply amazing. There's nothing better than when people share a little bit of themselves. Because … it somehow calms you down – it really does … (Informant 12)

The women gave examples of how the room and its audial and visual stimuli created opportunities for small talk and how they perceived this as calming. However, all the women in the study also expressed a need for guidance from the midwife, as they found themselves in unknown territory and overwhelmed by pain. One of the women spoke for many when describing her experience of such guidance*:*

… and I really needed that (…) I remember staring intensely into her eyes. She was so good at keeping my gaze and helping with breathing and everything. I was like … you know, I just surrendered myself … (Informant 14)

Our data indicate that in general the midwifes working in the alternative delivery room were successful in helping the women “surrender” themselves to the birth process and guide them into a sense of physical and psychological control. However, the amply spaced room and the physical facilities also facilitated another form of emotional support, as it allowed the midwife to retreat and offer a private space for the couple while remaining available to them. The women valued this occasional “professional retreat” for the opportunity it gave them to find strength through close connectedness with their partner while maintaining a feeling of safety and being cared for. The following quote is typical:

Really, the good thing was ... that she was there – and that she was there almost without us seeing her. Because she was sitting, like in a corner. (…) We could talk about all sorts of things (…) things she didn’t have to know. I think it was (…) we were really just – completely ourselves. And that was really the reason we felt as great as we did. (Informant 12)

The possibility for midwives to change between closeness and respectful distance seemed to increase the women’s confidence in their own abilities and sense of control. One woman explained this as:

I think she felt that I also could manage on my own … well I actually think it was such a good combination...She made it seem so natural so that we could feel we were the ones in charge…so we could feel comfortable.. yes well, it made me feel safe that I knew she was there anyway and that she was calm... it made me less stressful. (Informant 6)

The opportunity to create privacy in an unobtrusive way provides a key example of how the room enabled the midwives to adapt to different situations. Moreover, the environment seemed, in the majority of cases, to support or preserve the midwives ‘ability to provide a sensitivity to individual variations which is essential in ensuring patients’ experience of emotional support [24]. However, for few women the overall birth experience was overshadowed by their experience of insufficient emotional support from the midwives. They reported poor communication and misinterpretation of their needs, which led to anxiety and feelings of powerlessness. For instance, one woman described her frustration as:

I thought aren’t anyone going to help me? Is it not a Midwife’s job to say; Well then you take your breast and… There was NOTHING. I felt like the worst mother ever on earth ... I did not know how I was going to get her to breastfeed... and then I got afraid and you know... she was completely naked and s smooth. I felt so stupid…It ruined the whole experience of the birth for me. (Informant 4)

This seemed to emphasize what is known in a patient centered approach, that if patients’ emotional needs are not met, their usual resilience and ability to cope with stressful situation can be compromised [24]. The birth environment thus seems to be subordinate to the woman–caregiver relationship in the women’s assessment of their birth experiences.

#### Involvement of partner

##### Feeling equal

Overall, woman spoke positively about their partner’s role and found it very important for their own psychological state of mind during labor. They highly valued the opportunities offered by the room to interact with their partner in a natural, everyday manner. Many of them said it had promoted a sense of equality and strengthened the couple in working together, which they found had alleviated their anxiety. One woman’s description of the experience was typical:

... we were in there together, you know. It wasn’t all just about me … it was more like it was about both of us. When you’re sitting together, and I’m sitting the same way as him … not just lying in that damn bed. It makes a world of difference getting out of that bed. Because once you’re in it, you are a patient. And I didn’t feel like a patient when I walked around with him … or sat in that cushion area with him. You’re in it together. (Informant 8)

Our data clearly indicate that the sofa bed was the key feature and a major asset of the room. Most of the women talked excitedly about its role in their overall experience, even though it varied whether they had used it before or after birth (no participant used it for the birth although this was also possible). The sofa bed facilitated physical contact between the woman and her partner and strengthened her experience of intimacy and closeness with the partner, which the women saw as stimulating for their sense of partnership. A typical comment was:

… he was sitting right by my side on the sofa. I could feel his presence. He was … I could feel his legs ... it gave me a sense of security and tranquility. You know, we’re together in this, even if I’m doing the hard work. Anyway, having him alongside me, feeling him... was great for me. (Informant 13)

Maintaining intimacy and being equal was key to the women’s general experience of a high level of psychosocial support from their partner. In interpretation of our data, it seems that the women and her partner, in their way of being together in the room, must have experienced the physical environment to signal, encourage and allowing the partner, in some degree, to be involved in the care for the woman during childbirth. This is considered an essential aspect of patient-centered care which supports a family-centered approach [24] and clearly of great meaning to the women.

##### A space for the partner

The women expressed consistent consideration of their partner’s well-being during labor. Again, the sofa bed was highly valued as it increased the comfort of the partner while offering both a physical and psychological “space”. The relaxing effect allowed them to withdraw for rest when needed. This contributed to reducing the women’s concerns and stress stemming from her concern for the partner’s well-being. One woman explained it this way:

The corner made all the difference ... for the father too, and I actually felt that it was great for me as well. Because I felt like “Now I don’t have to worry about him”. I thought it was really nice ... because it means something. The person you’ve got with you in the room, you know, it’s someone you are extremely fond of … (Informant 12)

The sofa area afforded the partner an occasional retreat and the couple a chance to relax individually and in different ways without being separated. The women thus felt that the partner was always present when needed. The women seemed to experience that the sofa bed was an explicit sign that the environment acknowledged partner’s needs, which also were experienced supportive to the women’s needs.

#### Physical comfort

##### Positive distractions

For some of the women, the visual and auditory stimuli captured attention, which was helpful to their coping with labor and maintaining a sense of control. Several of the women described a feeling of being inside a bubble when coping with labor, as expressed by one woman:

It was just really nice for me, you know – the peace in just watching that [the projected images]. Somehow, I'm just inside this bubble. It was ... I really felt like I was in a bubble. It really helped me keep myself going, you know, maintaining the relaxed state I wanted to achieve … and it also turned out to be really good for me. (Informant 8)

Many of the women were able to recall their state of mind and body during labor, and they associated it with relaxation and joy. The stimuli elicited positive emotions in the birth environment, as they associated to important aspects of the couples’ daily lives. For instance, one woman said:

… [the landscape images] made him recall his experiences hunting (…) He kind of liked watching it. So we talked a lot about that – also with the midwife ... it was really nice… (Informant 10)

Many other distractions, such as childhood memories from the beach, were described by the women and their partners. Although they did not all enjoy such reminders of important aspects of their daily lives, they were all positive about the social interaction that the landscapes and sounds had given rise to.

The majority of the women furthermore emphasized the positive effect of the room lighting. The following quote is typical:

The light was so calming, I think. Like, there was no stress … you know, it was kind of more relaxing – a good feeling, with nothing to feel bad about, you know. We weren’t waiting, or afraid that something bad might happen, the way you might be in a hospital. (Informant 6)

In general, the warm, dimmed light in the room contributed to the women’s sense of comfort and being in a safe environment. Overall, they used and perceived the light and the distractions very differently. While some of the women did not register the audial and visual distractions during labor, they still emphasized the overall positive contribution of these stimuli in creating an positive ambience. A few women found that the audial and visual distraction compromised their ability to relax. However, as the women and their partners were given control of the sound, light, and the projected landscapes according to their current needs, stressful situations were avoided. Regardless of initiative tacking in the physical environment to support physical comfort, safeguarding of the woman and her partner’s autonomy is therefore a significant environmental factor in the support a patient centered approach.

##### Capturing the room

Many of the women were surprised to find that the ambience of the room gave them a sense of empowerment in actively and autonomously exploring the room and its facilities. A typical remark was:

I kind of felt I had to try capture the room in some way. Instead of just sitting and watching ... all of a sudden, I found it quite exciting and it made me feel comfortable … (Informant 8)

The tranquility and physical comfort provided by the room seemed to stimulate the women and their partners to move around freely to explore different spaces for relaxation and pain relief without feeling a need to ask the staff for permission or help. While many of the women found well-being in the bathtub, by walking around and adapting different positions close to their partner on the sofa bed, some also found the traditional hospital bed helpful. The environment seemed to support the recommendation in a patient centered approach to explicitly address and expand patients’ opportunity for individual-controlled options for pain relief [24]. However, all women gave birth in the traditional hospital bed, even though many expressed that they would rather have been giving birth in the bathtub or on the sofa bed. Many factors may be causing this, however as the women did not know the reason behind the decision to give birth in the traditional hospital bed, this could suggest that exploration of possibilities for alternative birth positions and communication regarding this depends on the guiding of the midwife.

## Discussion

A high level of patient-centered care is documented by the women’s predominantly positive perception of the alternative birth environment. Overall, our results therefore corroborate other research indicating that well-designed physical settings play an important role in making hospitals safer and more healing [12, 14, 29]. Our analysis has shown that women’s experiences of the physical environment in the alternative delivery room relates to several dimensions of the concept of patient centered care. Three main themes were developed: *Emotional support, Involvement of family and Physical comfort*, and especially four key factors within the environment contributed to the high level of patient-centered care.

Firstly, the ease of transition to the alternative and more home-like hospital setting seemed to strengthen the women’s experience of emotional as well as physical well-being. A possible explanation for this might be that comfortable and familiar environment is known to promote feelings of safety, confidence, and a sense of self and to have a strengthening effect on women’s physiological functioning and emotional well-being [11]. The environment therefore seemed to convey an atmosphere of dignity, respect, and sensitivity toward the women’s psychological needs, which are an essential element of patient centered care [24] and could be a facilitator of progress during birth [13].

Secondly, the women’s partners were also offered physical and psychological space by the room, hereby relieving the well-known worries of repercussions for relatives often experienced by patients [24]. As described by [13], sharing the experience of labor and birth with their partner is extremely important for most women. While the value of the partner’s presence and support for women’s birth experiences has been documented in other studies [30], it has also been suggested that this factor slows down the process of labor as women may find it harder to relax with her partner by her side [13]. However, in our study the women experienced the sofa bed as a significant feature supportive to relaxation of both the women and her partner.

The experienced possibility offered by the facilities to their partners as providers of emotional and physical support during labor and birth was also of great importance to our informants. In order to support real involvement of families, this explicit encouragement within the environment of the family’s needs should however also entail health professional to seek a deeper exploration of the partners individually expressed needs [24]. The partners experience is however not within the scope of this study focusing on the women’s experience.

Thirdly, most women found that the environment helped them obtain physical comfort and encouraged active behavior, which may offer psychological benefits in their coping with labor, relaxation, and feeling in control. As shown by Raynor and England [13], the progress of labor and birth is facilitated by having access to and control over a variety of ways of obtaining physical comfort during labor and birth.

Literature has also described how psychological benefits of positive distractions helps people to attend to the stimuli rather than own discomfort and anxiety [14, 29, 31]. In our study women most women also found joy and relaxation in the positive distractions. However, most importantly the autonomy offered with respect to these physical features avoided potential stressful situations. According to her needs, the women experienced that she overall was free to seek the support of her partner or the midwife, or to be left alone, a feature of patient-centered care which also is considered to offer protection of patients’ autonomy and promote family centered care [24]. Safeguarding of woman and partner’s autonomy is therefore a significant environmental factor, which also has been shown to support progress in labor [13] and should therefore advantageously be preserved and thought into future hospital settings and quality of care.

Finally, the alternative delivery room overall appeared to elicit positive and supportive relationships between the couples and their midwife, enabling the midwives to explore and respect the women’s psychological needs, which seem to have amplified the women’s positive experiences of the birth environment. Our study thereby suggest that both the environment and midwives play an in important role in women achieving a physical comfort and a sense of control. This is also supported by studies, showing that women who trust the midwife’s ability to control labor tend to experience this as pain reducing; such factors are known to have neurobiological positive effects on natural birth [15, 32].

Even though the essential findings within the women’s experiences were found to be related to the three main themes mentioned above, other dimension of patient centered care are also indirectly represented in the women’s experience e.g. respecting patients values and needs, integration of care and information and communication. However, understandings of the dimension of transition and continuity were not found in interpretation of the women’s experiences.

### Strengths and limitations

The individual, in-depth interviews with their thematic focus have produced rich data offering new insights into women’s experiences of birth in an alternative delivery room and its ability to support patient-centered care. Data saturation was achieved through the 14 interviews, which is in line with other authors reporting data saturation after twelve interviews [33]. We did however not fully achieve our aim for great variation in the study participants’ key characteristics, and thus in their perspectives and experiences. For instance, none of them was single or young mothers, born outside Denmark, or in a disadvantaged social position. Our sample does, however, closely reflect the characteristics of the women already enrolled in the RCT on medical birth outcomes, from which the informants were recruited. A greater variety among participant characteristics may have contributed to further data variety, and our finding may be less transferable to single women or women/couples with another cultural background or less advantageous life circumstances. Another potential limitation lies in the great professional and public interest in the alternative delivery room prior to the study, which may have influenced the women’s preunderstandings and expressed views. The interviewer addressed this by constant awareness and ensuring adequate depth of the interviews. Furthermore, the reflexivity of the interviewer’s role and preconception was discussed by the authors at all stages of the research process.

### What this study adds

The findings have added to the evidence on the positive influence of hospital environment design on patients’ psychological and physical well-being and thereby the psychosocial outcomes of care. Our study furthermore offers new insights into how an alternative delivery room inspired by the principles of healing architecture and Snoezelen can help encourage service providers to deliver patient-centered care, on which many hospitals and health care institutions base their practices.

### Implications for practice

Our findings support the use of the principles of healing architecture and Snoezelen in the development of evidence based birthing environments. We consider that they apply also to rooms used for preliminary examination. Special attention should be given to ensuring space for the woman’s partner, the couple’s physical and emotional interaction, and in upholding women’s autonomy in the use of facilities to increase relaxation and physical comfort. However, ensuring and maintaining one-on-one care appears to be crucial, as the women saw the midwife–couple relationship and the psychosocial support offered by the midwife as a key factor in their birth experience.

## Conclusion

In general, the women were found to have had very positive experiences with giving birth in the alternative birth environment. The environment appeared to encompass several dimensions of the concept of patient-centered care. Overall, the physical environment accommodated many of the women’s individual, psychological needs, e.g. by facilitating easy transition to hospital which reduced stress and anxiety. The environment furthermore seemed to provide opportunities for physical comfort and relaxation, which helped the women cope and their sense of being in control. The women placed great emphasis on the ability of the alternative delivery room to support not only their own needs but also their partner’s. They likewise stressed the psychosocial support and the interaction with the partner. However, attention should be given to ensure and maintain one-on-one care as the midwife’s psychosocial support appeared to be key to women’s birth experiences and inseparable from the alternative birth environment. All these factors appeared to be highly interdependent. Our findings thus support the development of patient-centered birth environments in future hospital design to promote the physical and psychological wellbeing of the woman and her partner.

## Data Availability

The datasets generated and/or analyzed during the current study are not publicly available due to considerations on privacy of participants but are available from the corresponding author on reasonable request.

## Abbreviation

RCT: Randomized Controlled Trial
